# Expression of high p53 levels in colorectal cancer: a favourable prognostic factor

**DOI:** 10.1038/sj.bjc.6690660

**Published:** 1999-09

**Authors:** E Adrover, M L Maestro, M T Sanz-Casla, V del Barco, J Cerdán, C Fernández, J L Balibrea

**Affiliations:** Department of Oncology, Hospital ‘Virgen de la Luz’, Donantes de Sangre Avenue s/n., Cuenca, 16002, Spain; Departments of; Tumour Biology (Laboratory), Surgery (II Department) and Epidemiology, Hospital Clínico ‘San Carlos’, Madrid, Spain

**Keywords:** colorectal cancer, p53, immunoassay, luminometric, prognosis, stage III cancer

## Abstract

The expression of p53 protein was examined in a series of 111 colorectal cancer adenocarcinomas with a long follow-up. A quantitative luminometric immunoassay (LIA) was used for the measurement of wild-type and mutant p53 protein in extracts from colorectal tumour cytosols, p53 being detected in 42% of the samples (range 0.0–52 ng mg^−1^). Using an arbitrary cut-off value of 2.7 ng mg^−1^, 25% of the tumours were classified as manifesting high p53 levels. There was no association of p53 expression with patient age, sex, serum preoperative carcinoembryonic antigen (CEA) levels, tumour site and size, nodal status or TNM stage. Significant and independent correlation was found to exist between high p53 levels and prolonged disease-free survival (*P* = 0.05) at a median follow-up of 60 months. This survival advantage was most apparent among stage III cancer patients. The results from this study would suggest that expression of high p53 levels appear to be useful in selecting a group of colorectal cancer patients with a better prognosis. © 1999 Cancer Research Campaign


*British Journal of Cancer* (1999) **81**(1), 122–126
© 1999 Cancer Research Campaign
Article no. bjoc.1999.0660

### Expression of high p53 levels in colorectal cancer: a favourable prognostic factor

**E Adrover[1], ML Maestro[2], MT Sanz-Casla[2], V del Barco[2], J Cerdán[3], C Fernández[4] and JL Balibrea[3]**

[1]Department of Oncology, Hospital 'Virgen de la Luz', Donantes de Sangre Avenue, s/n. 16002 Cuenca, Spain; Departments of [2]Tumour Biology (Laboratory), [3]Surgery (II Department) and [4]Epidemiology, Hospital Clínico 'San Carlos', Madrid, Spain

**Summary** The expression of p53 protein was examined in a series of 111 colorectal cancer adenocarcinomas with a long follow-up. A quantitative luminometric immunoassay (LIA) was used for the measurement of wild-type and mutant p53 protein in extracts from colorectal tumour cytosols, p53 being detected in 42% of the samples (range 0.0–52 ng $mg^{-1}$). Using an arbitrary cut-off value of 2.7 ng $mg^{-1}$, 25% of the tumours were classified as manifesting high p53 levels. There was no association of p53 expression with patient age, sex, serum preoperative carcinoembryonic antigen (CEA) levels, tumour site and size, nodal status or TNM stage. Significant and independent correlation was found to exist between high p53 levels and prolonged disease-free survival ($P = 0.05$) at a median follow-up of 60 months. This survival advantage was most apparent among stage III cancer patients. The results from this study would suggest that expression of high p53 levels appear to be useful in selecting a group of colorectal cancer patients with a better prognosis.

**Keywords:** colorectal cancer; p53; immunoassay; luminometric; prognosis; stage III cancer

The relationship between molecular abnormalities and neoplasm has been extensively reviewed and there is strong evidence that abnormalities of p53 gene represent the most common molecular change in human cancer. Such abnormalities can be detected in a number of ways. Several prior studies have revealed p53 protein expression via immunohistochemistry (IHC) in 42–69% of colorectal cancers (Scott et al, 1991; Auvinen et al, 1994). However, the prognostic value of this protein remains to be defined, probably due to variability of detection and retrieval systems (Wynford-Thomas, 1994). To be clinically useful, prognostic markers should be accessible to analysis with simple and reproducible procedures appropriate for routine use. We have adapted the recently developed luminometric immunoassay (LIA) (Borg et al, 1995), to quantitate p53 protein in archival colorectal cytosols.

The purpose of the present study was: (1) to evaluate the relationship between p53 overexpression and clinicopathological data and (2) to assess the value of p53 as a biological marker of prognosis within each TNM class in a series of patients resected for colorectal cancer with a long follow-up.

### PATIENTS AND METHODS

### Patients

A series of 111 patients underwent surgical resection for primary colorectal adenocarcinoma at the II Department of Surgery, University Hospital 'San Carlos', Madrid, between 1990 and 1992. Each patient is regularly followed up at 6-monthly intervals for a minimum of 5 years. Cases in which resections have been performed for metachronous carcinoma, carcinoma arising in familial adenomatous polyposis and ulcerative colitis are excluded. None of the patients had received preoperative radiotherapy or chemotherapy. Since 1992, stage III patients younger than 70 received adjuvant chemotherapy with 5-fluorouracil (5-FU) and leucovorin according to the prevailing protocol (four patients). Clinical staging was done on the basis of the TNM classification. Survival time was calculated from the date of surgery to the date of death or last follow-up, with times censored for patients dying of causes unrelated to colorectal cancer and those surviving. Median follow-up was 5 years.

*Received 20 November 1998*
*Revised 4 February 1999*
*Accepted 11 March 1999*

*Correspondence to:* E Adrover

### Tissue specimens

Sections from the colorectal adenocarcinoma and normal mucosa at the proximal/distal resection margins were obtained at surgical resection. These specimens were stored in liquid nitrogen and, prior to being pulverized in the frozen state and homogenized for the preparation of cytosols for protein measurement, cryostat sections were evaluated; all tumour samples used contained more than 80% tumour cells.

### Luminometric assay

The LIA is based on a combination of two monoclonal antibodies, Ab 1801 and DO 1, which detect both wild-type and mutant p53 protein in a sandwich-type assay. The Ab1801, which is immobilized onto a solid phase, is used for catching. Ab DO 1, labelled with a chemiluminescent compound (ABEI), is used for detection. The immunoassay was performed by incubating either 100 µl of p53 standard, controls or tumour cytosols, together with 100 µl of the ABEI-conjugate in pre-coated tubes. After incubation for 18 h at room temperature, unbound reagents were removed by washing

**Table 1** Relationship between p53 protein levels in colorectal tumours and clinicopathological variables

| Variable | *n* (%) | p53 ≥ 2.7 ng mg$^{-1}$ | p53 < 2.7 ng mg$^{-1}$ | *P* |
|---|---|---|---|---|
| Age | 111 range: 31–89 (100) | 27 (25) | 84 (75) | |
| | Mean 66 years | Mean 67 years | Mean 66 years | 1 |
| Sex | Male: 54 (49) | 15 (27.8) | 39 (72.2) | |
| | Female: 57 (51) | 12 (21) | 45 (79) | 0.51 |
| | I: 14 (12.6) | 3 (21.4) | 11 (78.6) | |
| Stage | II: 50 (45) | 11 (22) | 39 (78) | |
| | III: 30 (27) | 10 (33.3) | 20 (66.7) | 0.6 |
| | IV: 17 (15.3) | 3 (17.6) | 14 (82.4) | |
| T status | T1–T2: 17 (15) | 5 (29.4) | 12 (70.6) | |
| | T3–T4: 94 (85) | 22 (23.4) | 72 (76.6) | 0.6 |
| N status | N0: 65 (58.6) | 15 (23) | 50 (77) | |
| | N+: 46 (41.4) | 12 (26) | 34 (74) | 0.8 |
| | Right colon: 28 (25) | 7 (25) | 21 (75) | |
| Site | Left colon: 36 (33.4) | 8 (22.2) | 28 (77.8) | 0.94 |
| | Rectum: 47 (39.6) | 12 (25.5) | 35 (74.5) | |
| CEA | <5 ng ml$^{-1}$: 42 (42) | 7 (16.7) | 35 (83.3) | |
| | ≥5 ng ml$^{-1}$: 58 (58) | 18 (31) | 40 (69) | 0.16 |

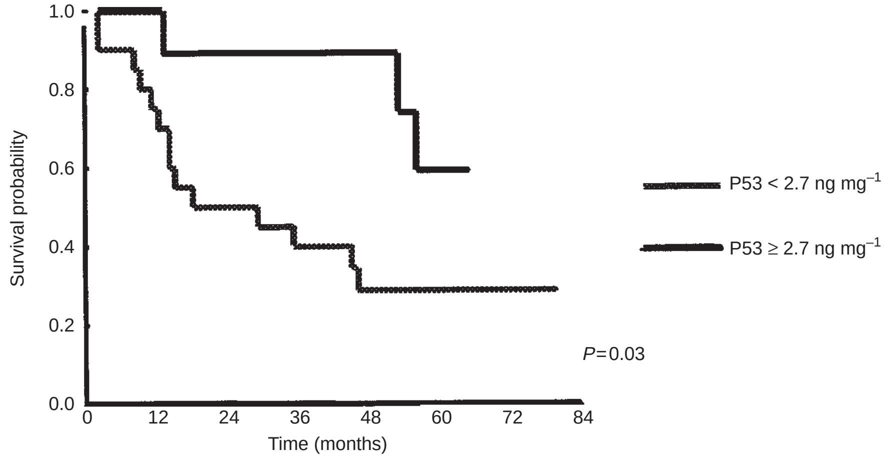

**Figure 1.** Stage III colorectal cancer patients. Disease-free survival curve according to p53 levels

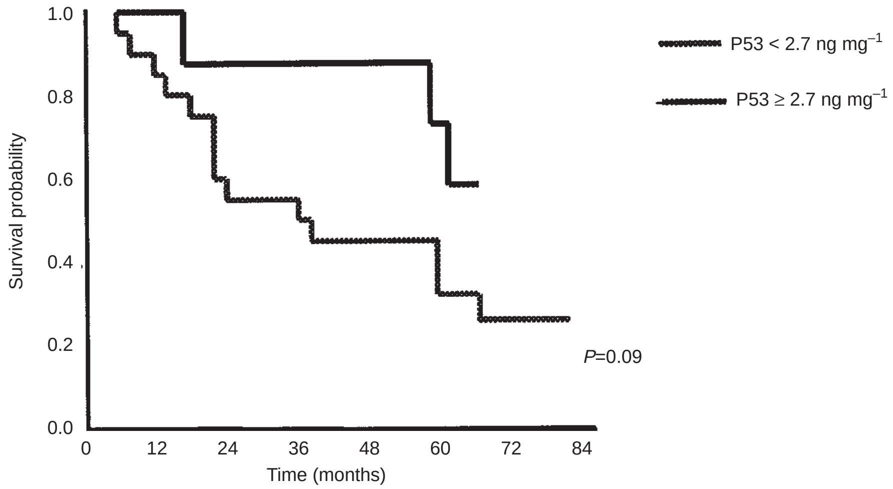

**Figure 2.** Stage III colorectal cancer patients. Overall survival curve according to p53 levels

*© 1999 Cancer Research Campaign*

**Table 2** p53 immunohistochemical assays and prognosis in colorectal cancer

| Reference | Patients | Follow-up | p53 poor prognostic factor | Multivariate analysis Yes/No and other comments |
|---|---|---|---|---|
| Sun 1993 | 293 | 5 years | Y | MVA – yes. Nuclear and cytoplasmic p53 associated with adverse prognosis in I-III stages |
| Yamaguchi 1992 | 100 | 6–48 months | Y | MVA – yes. 3-year survival significantly worse for p53-expressing cases |
| Bell 1993 | 100 | 34 months | N | No MVA. No relationship of p53 and overall survival |
| Yamaguchi 1993 | 203 | 5 years | Y | No MVA. P53-positive cases associated with adverse survival |
| Auvinen 1994 | 144 | 9 years | Y | No MVA. Overall survival reduced in p53 positive cases |
| Nathanson 1994 | 84 | > 5 years | N | MVA – yes. No relationship of p53 and overall survival identified in stage II |
| Zeng 1994 | 107 | 62 months | Y | MVA – yes. Study conducted in stage III–IV patients with low CEA levels |
| Mulder 1995 | 109 | > 7 years | N | MVA – yes. P53 not an independent marker of prognosis in all stages. |
| Ofner 1995 | 109 | 79 months | N | No MVA. p53 expression not an independent marker of prognosis |
| Kressner 1996 | 294 | 4.5 years | N | MVA – yes. No relationship of p53 and overall survival identified in all stages |
| Poller 1997 | 250 | 4.3 years | N | MVA – yes. No relationship of p53 and overall survival identified in stage I–III tumours |

the tubes three times with 2 ml 0.9% sodium chloride. The chemiluminescent reaction was initiated by the sequential addition of 300 µl microperoxidase solution, immediately followed by measurement of the chemiluminescent counts in a luminometer. The p53 protein contents of the samples were determined from the standard curve plotting the chemiluminescent response (in relative light units (RLU)) against the standard concentrations of p53 protein. The detection limit was 0.01 ng p53 per ml sample. p53 concentration in the cytosols is expressed as ng $mg^{-1}$ cytosolic protein, the cytosol protein concentration being in the range 0.5–4 ng $ml^{-1}$.

### Statistical analyses

To evaluate the relationship between p53 expression and tumour behaviour, different cut-off values were tested and then 2.7 ng $mg^{-1}$ was selected as the cut-off value that yielded the best discrimination of patients with good vs poor prognosis. Statistical significance of differences was determined by the $\chi^2$ or Fisher and the Student's *t*-test or analysis of variance (ANOVA). Survival rates were estimated by the Kaplan–Meier method and tested for significance by the Breslow exact test, while multivariate comparisons were conducted using the Cox regression analysis. Stratified analysis was used. Relative risks and their 95% confidence intervals (CI) were calculated with the use of the estimated regression coefficients and their standard errors. A *P*-value of < 0.05 was considered significant. Analysis was performed using the SPSS 7.5 statistical software.

### RESULTS

Survival data, available on all cases, showed a 7-year specific overall survival of 49% (median 57 months). During follow-up, 37 patients had a relapse and 54 died from cancer-related deaths. Fifteen patients died from other causes. The LIA revealed p53 overexpression only in 47 neoplastic samples (42.3%), while the adjacent colonic mucosa was constantly negative. p53 values in tumour cytosols ranged between 0 and 52 ng $mg^{-1}$ protein, 75% of the samples < 2.7 ng $mg^{-1}$, with a mean value of 4.4 ng $mg^{-1}$ protein. With regard to patient and tumour characteristics there were no significant relationship between p53 levels and tumour site, age, serum preoperative carcinoembryonic antigen (CEA) levels, stage, nodal status or tumour invasion through the bowel wall. Detailed information on patient and tumour characteristics is given in Table 1 for each subset analysed.

### Stratified analyses

Since tumour stage at diagnosis is the most powerful indicator of clinical outcome in colorectal cancer patients, p53 expression prognostic significance was assessed within each TNM class. Only patients with stage III cancer whose tumours expressed high p53 levels had longer disease-free survival times than patients with tumours lacking or with low-expressing LIA-detectable p53 protein (59.3% and 28.6% respectively) at a median follow-up of 60 months ($P = 0.03$). A longer overall survival (58.3% and 30%) was also observed, although this finding did not reach statistical significance ($P = 0.09$) (Figures 1 and 2).

### Multivariate analyses

In multivariate analyses for relapse-free survival, including standard prognostic factors as covariates, p53 level < 2.7 ng $mg^{-1}$ was an independent factor for predicting the rate of relapse, with an adjusted relative hazard rate of 2.13 (CI 0.89–5.07) ($P = 0.05$). This association was independent of the TNM stage. However, p53 overexpression did not reach statistical significance for overall survival.

© 1999 Cancer Research Campaign

### DISCUSSION

A sensitive and quantitative LIA has been developed for the measurement of wild-type and mutant p53 in extracts from tumour tissue. The p53–LIA has been performed previously on breast tumour cytosols (Borg et al, 1995) where it yielded reliable estimates of p53 expression as compared with IHC analysis. This novel immunoassay, although relatively time-consuming, allows an exact quantification of p53 expression in a way that is superior to IHC. In our series, p53 protein was detected in 42% of the tumour samples; this is well in line with previously reported p53 IHC overexpression in colorectal carcinomas, ranging from 42 to 69% (Scott et al, 1991; Auvinen et al, 1994). We found no correlation between p53 levels, tumour stage, site, serum preoperative CEA levels, bowel wall invasion and nodal status. Similar results have been reported previously (Scott et al, 1991; Starzynska et al, 1992; Bell et al, 1993; Nathanson et al, 1994; Mulder et al, 1995; Poller et al, 1997). At least 30 investigations have dealt with the prognostic significance of p53 aberrations in colorectal adenocarcinoma, providing contrasting results (Viale, 1997). In the vast majority of these studies, IHC was used to document an abnormal accumulation of p53 protein in neoplastic cells. The variables related to the staining protocols and scoring systems are so numerous that it is almost impossible to compare the results of different studies. Table 2 lists some of the most relevant published data on the prognostic value of p53 IHC expression in colorectal cancer; most would suggest that expression of p53 is an adverse prognostic factor. However, of the seven investigations using multivariate analysis, three documented a significant inverse correlation of p53 accumulation with patient survival, whereas four did not.

To clarify the influence of p53 overexpression on long-term survival in colorectal cancer patients, we performed LIA based on a combination of two monoclonal antibodies, Ab1801 and DO 1, which detects both wild-type and mutant p53 protein. No difference in survival was found between p53-positive and -negative tumours in the whole series, so we used the cut-off value of 2.7 ng $mg^{-1}$ for survival analysis. The most important finding was that high p53 level in the tumour cytosols was associated with favourable disease-free survival ($P = 0.45$) in all stages, but not significantly by univariate analysis. This survival advantage was most apparent among stage III cancer patients. The multivariate analyses confirmed that p53 expression was an independent prognostic factor for time to progression when associations between variables were accounted for ($P = 0.05$). As far as we are aware, only two previous studies have directly correlated p53 accumulation with prolonged survival in stage II–III colorectal cancer patients (Soong et al, 1997; Ahnen et al, 1998); interestingly, the survival advantage was more significant for stage III patients. In two previous studies, this association appeared to become more significant as the estimated percentage of cells staining positively increased (Dix et al, 1994*a*); it was shown that IHC detection of p53 protein does not always indicate the existence of an underlying mutation (Dix et al, 1994*b*). Other studies on non-small-cell lung cancer (Lee et al, 1995), breast cancer (Sjögren et al, 1996) and testis cancer (Eid et al, 1997) have observed the same unexpected favourable prognosis for patients with high p53 immunostaining. However, the organ and tissue of origin of the tumour seems to have a crucial role in determining overall p53 function.

p53 overexpression is the end point of the action of many different stabilizing mechanisms, which ultimately lead to p53 accumulation. LIA detects this overexpressed p53, without indicating the mechanisms by which accumulation occurs. In the past, it was assumed that only the mutant p53 protein, not the wild-type, would be detected by IHC. This view was based on the observation that the wild-type p53 protein degrades rather rapidly, with a short half-life of 6–20 min, compared with most mutant p53 proteins, which have longer half-lives that range from 4 to 12 hours (Tominaga et al, 1992). However, clinical investigators have observed that positive immunostaining does not always mean gene mutation (Battifora, 1994). In addition, increases in wild-type p53 protein have been well documented in association with DNA damage repair (Kastan et al, 1991) and after UV irradiation of normal human skin (Campbell et al, 1993). Furthermore, not all p53 mutations translate to positive immunostaining. Point mutations leading to a stop codon, abnormal mRNA splicing, or gross deletion or insertions are well-known cases in which immunostaining is negative despite gene mutations. These data are of particular importance, because they imply that low levels of LIA are not always indicative of a normal p53 gene status. More importantly, accumulating data indicate that not all mutant p53 proteins lose the normal p53 functions, and the function of the mutant p53 gene product is variable depending on the mutation locus (Cho et al, 1994; Levine et al, 1994). Recent studies have associated a poor prognosis for colon cancer with specific p53 mutation (Børresen-Dale et al, 1998). The higher rates of elevated p53 levels we have detected in stage III colorectal tumours could explain such an observed better prognosis, but as we have not screened our population of patients for p53 gene mutations, we cannot rule out the genetic factors responsible for this. Whether the tumours of this subset of patients have normal p53 or mutations which retain wild-type function, rendering the tumours less aggressive, remains to be studied.

In summary, it appears from our study that p53 protein overexpression may tell us more about the functional status of the p53 control pathway than the presence of a mutation within the gene. While the exact relationship between p53 gene mutation and protein overexpression in tumours is determined, quantification of p53 protein levels may be a useful parameter to optimize treatment for patients with completely resected colorectal cancer.

### REFERENCES

Ahnen DJ, Feigl P, Quan G, Fenoglio-Preiser C, Lovato L, Bunn PA Jr, Stemmerman G, Wells JD, MacDonald JJ and Meyskens FL Jr (1998) Ki-*ras* mutation and p53 overexpression predict the clinical behaviour of colorectal cancer: a Southwest Oncology Group study. *Cancer Res* **58**: 1149–1158

Auvinen A, Isola J, Visakorpi T, Koivula T, Virtanen S and Hakama M (1994) Overexpression of p53 and long term survival in colon carcinoma. *Br J Cancer* **70**: 293–296

Børresen-Dale AL, Lothe RA, Meling GI, Hainault P, Torlaiv OR and Skovlund E (1998) TP53 and long-term prognosis in colorectal cancer, mutations in the L3 Zn-binding domain predict poor survival. *Clin Cancer Res* **1**: 203–210

Battifora H (1994) p53 immunochemistry: a word of caution. *Hum Pathol* **25**: 435–436

Bell S, Scott N, Cross D, Sagar P, Lewis FA, Blaire GE, Taylor GR, Dixon MF and Quirke P (1993) Prognostic value of p53 overexpression and c-ki-*ras* gene mutations in colorectal cancer. *Gastroenterology* **104**: 57–64

Borg A, Lennerstand J, Stenmark-Askmalm M, Ferno M, Bristors A, Ohrvik A, Stal O, Killander D, Lane D and Brundell J (1995). Prognostic significance of p53 overexpression in primary breast cancer: a novel luminometric immunoassay applicable on steroid receptors cytosols. *Br J Cancer* **71**: 1013–1017

Campbell C, Quinn AG, Angus B, Farr PM and Rees JL (1993). Wavelength-specific patterns of p53 induction in human skin following exposure to UV radiation. *Cancer Res* **53**: 2697–2699

© 1999 Cancer Research Campaign 

Cho Y, Gorina S, Jeffrey PD and Pavletich NP (1994). Crystal structure of a p53 tumour suppresser DNA complex: understanding tumorigenic mutations. *Science* **265**: 346–355

Dix B, Robbins P, Carello A, House A and Iacopetta B (1994*a*). Comparison of p53 gene mutation and protein overexpression in colorectal carcinomas. *Br J Cancer* **70**: 585–590

Dix BR, Robbins P, Soong R, Jenner D, Hove AK and Iacopetta BI (1994*b*). The common molecular genetic alterations in Dukes' B and C colorectal carcinomas are not short-term prognostic indicators of survival. *Int J Cancer* **50**: 747–751

Eid H, Van der Looij M, Institoris E, Géczi L, Bodrogi I, Olah E and Bak M (1997). Is p53 expression, detected by immunohistochemistry, an important parameter of response to treatment in testis cancer? *Anticancer Res* **17**: 2663–2670

Elledge RM and Allred DC (1994). The p53 tumour suppressor gene in breast cancer. *Breast Cancer Res Treat* **32**: 39–47

Kastan MB, Onyekwere O, Sidransky D, Vogelstein B and Craig RW (1991). Participation of p53 protein in the cellular response to DNA damage. *Cancer Res* **51**: 6304–6311

Kressner U, Lindmark G, Gerdin B, Pahlman L and Glimelius B (1996) Immunohistological p53 staining is of limited value in the staging and prognostic prediction of colorectal cancer. *Anticancer Res* **16**: 951–958

Lee JS, Yoon A, Kalapurakal SK, Ro J, Lee J, Tu N, Hittleman WN and Hong WK (1995). Expression of p53 oncoprotein in non-small cell lung cancer: a favourable prognostic factor. *J Clin Oncol* **13**: 1893–1903

Levine AJ, Perry ME, Chang A, Silver A, Dittmer D, Wu M and Welsh D (1994). The 1993 Walter Hubert Lecture: the role of p53 tumour suppresser gene in tumoriginesis. *Br J Cancer* **69**: 409–416

Mulder JWR, Baas IO, Polak MM, Goodman SN and Offerhans GJA (1995). Evaluation of p53 protein expression as a marker for long term prognosis in colorectal carcinoma. *Br J Cancer* **71**: 1257–1262

Nathanson SD, Linden MD, Tender P, Zarbo RJ, Jacobsen G and Nelson LT (1994) Relationship among p53, stage and prognosis of large bowel cancer. *Dis Colon Rectum* **37**: 527–534

Ofner D, Maier H, Riedmann B, Holzberger P, Nogler M, Totsch M, Bankfalvi A, Winde G, Bócker W and Schmid KW (1995) Immunohistochemically detectable p53 and mdm-2 oncoprotein expression in colorectal carcinoma: prognostic significance. *J Clin Pathol Mol Pathol* **48**: 12–16

Poller DN, Baxter KJ and Sepherd NA (1997) P53 and Rb1 protein expression: are they any prognostically useful in colorectal cancer? *Br J Cancer* **75**: 87–93

Scott N, Sagar P, Stewart J, Blair G, Dixon M and Quirke P (1991) P53 in colorectal cancer: clinicopathological correlation and prognostic significance. *Br J Cancer* **63**: 317–319

Sjögren S, Inganäs M, Norberg T, Lindgren A, Nordgren H, Holmberg L and Bergh J (1996) The p53 gene in breast cancer: prognostic value of complementary DNA sequencing versus immunohistochemistry. *J Natl Cancer Inst* **88**: 173–182

Soong R, Grieu F, Robbins P, Dix B, Chen D, Parsons R, House A and Iacopetta B (1997) P53 alterations are associated with improved prognosis in distal colonic carcinomas. *Clin Cancer Res* **3**: 1405–1411

Starzynska T, Bromley M, Ghosh A and Stern PL (1992) Prognostic significance of p53 overexpression in gastric and colorectal carcinomas. *Br J Cancer* **66**: 558–562

Sun XF, Carstenten JM, Stal O, Zhang H, Nilsson E, Sjödahl R and Nordenskjöld B (1993) Prognostic significance of p53 expression in relation to DNA ploidy in colorectal adenocarcinoma. *Virch Arch A Pathol Anat* **423**: 443–448

Tominaga O, Hamelin R, Remvikos Y, Salmon RJ and Thomas G (1992) p53 from basic research to clinical applications. *Crit Rev Oncog* **3**: 257–282

Viale G (1997) Prognostic and predictive value of p53 aberrations in tumours of the gastrointestinal tract and pancreas. In: *Prognostic and Predictive Value of p53*, Vol. 1, Klijn JGM (ed), pp. 131–141. ESO Scientific Updates, Elsevier: Amsterdam

Wynford-Thomas D (1994) P53 in tumour pathology: can we trust immunocytochemistry? *J Pathol* **166**: 329–330

Yamaguchi A, Kurosaka Y, Fushida S, Kanno M, Yonemura Y, Miwa K and Miyazaki I (1992) Expression of p53 protein in colorectal cancer and its relationship to short-term prognosis. *Cancer* **70**: 2778–2784

Yamaguchi A, Nakagawa G, Kurosaka Y, Nishimura G, Yonemura Y and Miyazakii T (1993) P53 immunoreaction in endoscopic biopsy specimens of colorectal cancer and its prognostic significance. *Br J Cancer* **68**: 399–402

Zeng ZS, Sarkis AS, Zhang ZF, Klimstra DS, Charytonowicz E, Guillén JG, Cordón-Cardo C and Cohen AM (1994) P53 nuclear overexpression: an independent predictor of survival in lymph node-positive colorectal cancer patients. *J Clin Oncol* **12**: 2043–2050

 *© 1999 Cancer Research Campaign*
